# Dengue Fever Complicated by Pneumonia in Pregnancy: A Case Report

**DOI:** 10.7759/cureus.73608

**Published:** 2024-11-13

**Authors:** Hasan Bani Hani, Sara Ibrahim, Mayar Esmail, Shamsa Waleed, Saria Gouher

**Affiliations:** 1 Internal Medicine, University Of Sharjah, Sharjah, ARE; 2 Pediatrics, American Hospital Dubai, Dubai, ARE; 3 Pediatrics, Dubai Health Authority, Dubai, ARE; 4 Internal Medicine, American Hospital Dubai, Dubai, ARE

**Keywords:** dengue fever (df), dengue thrombocytopenia, infection in pregnancy, pneumonia, superimposed infections

## Abstract

Dengue fever, a mosquito-borne viral disease, was the most prevalent arthropod-borne illness globally and posed a significant public health challenge due to its increasing incidence and potential complications. While most patients recovered within one to two weeks, some cases progressed to severe dengue, requiring hospitalization. This case report described a 39-year-old pregnant woman at 27 weeks gestation who developed severe dengue fever complicated by bacterial pneumonia, leading to ICU admission. She initially presented with fever, tachycardia, and vomiting, and her diagnosis was confirmed by polymerase chain reaction (PCR). Due to worsening respiratory symptoms, she was treated with oxygen and antibiotics, which stabilized her condition for discharge. This case underscored the need for vigilance in managing dengue during pregnancy, as secondary bacterial infections, though uncommon, could complicate treatment. Prompt recognition and early antibiotic intervention in high-risk patients were crucial for improving outcomes.

## Introduction

Dengue, a virus transmitted by mosquitoes, was the most widespread viral disease transmitted by arthropods worldwide and posed a significant public health concern due to its rising incidence and potential complications. In 2019, around 129,972 cases were reported in the Middle East and North Africa (MENA) region alone, with 366 deaths [1]. Dengue viruses were primarily transmitted to humans through the bite of an infected Aedes mosquito [2]. Typical symptoms of dengue include high fever, headaches, body aches, nausea, and a rash. While most individuals recovered within one to two weeks, some cases progressed to severe dengue, requiring hospitalization [3]. Dengue fever in pregnancy disrupts critical physiological systems, elevating risks of complications such as placental abruption, preterm birth, and fetal distress. Management paralleled that in non-pregnant patients but required vigilant monitoring of fluid balance and hemodynamics to prevent severe complications like DHF and DSS, which could lead to multi-organ failure and miscarriages, particularly in early pregnancy. Vertical transmission, while not typically causing long-term fetal effects, necessitated monitoring [4]. This complexity was illustrated in the following case report of a 39-year-old pregnant woman at 27 weeks gestation who developed dengue fever with subsequent pneumonia, necessitating ICU admission for comprehensive care.

## Case presentation

A 39-year-old pregnant woman presented to the ER on Day 1, at 27 weeks gestation, with maternal pyrexia, tachycardia, vomiting, and fetal tachycardia. Notably, her husband had recently been hospitalized for dengue fever. She subsequently developed fever, nausea, vomiting, abdominal pain, fatigue, and joint pain, with laboratory findings indicating low hemoglobin and platelets, along with a positive PCR for dengue. Admission to the ICU was warranted due to fever and tachypnea, necessitating oxygen supplementation, intravenous paracetamol, and fluids. Additionally, the patient reported recent exposure to mosquitoes and experienced non-bloody vomiting. Upon admission, she was conscious with a Glasgow Coma Scale score of 15/15. Her maximum recorded temperature was 38.8°C, and her blood pressure was measured at 102/59 mmHg. She had a BMI of 38.93 with bilateral lower limb swelling. Subsequent examinations revealed a faint pink rash and bilateral wheezing.

**Figure 1:**
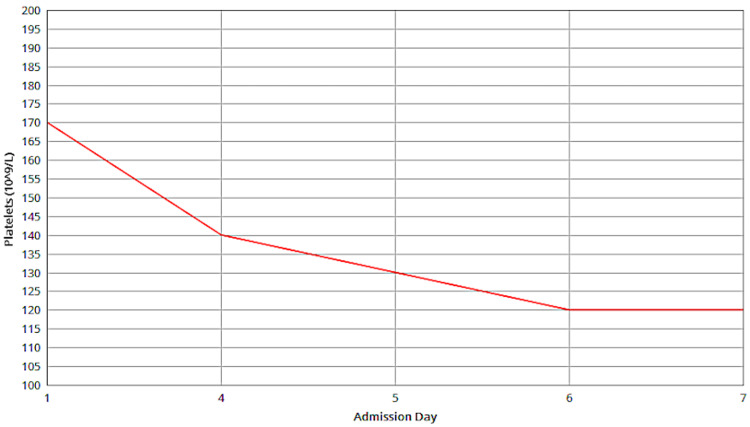
Platelets Depicts the platelet count trend from Day 1, to Day 7, showing a gradual decline over this period.

Following ICU admission, the patient exhibited stable vital signs, although a persistent fever and wheezing were observed. Despite improved wheezing post-nebulization, diminished breath sounds and new symptoms of chest pain, tachypnea, and bilateral basal crackles emerged, prompting further monitoring and management. As an inpatient on Day 2, a complete blood count revealed a white cell count of 5.3×10⁹/L. The platelet count was 142×10⁹/L, showing a gradual decline over the admission period, as shown in Figure 1.

**Figure 2:**
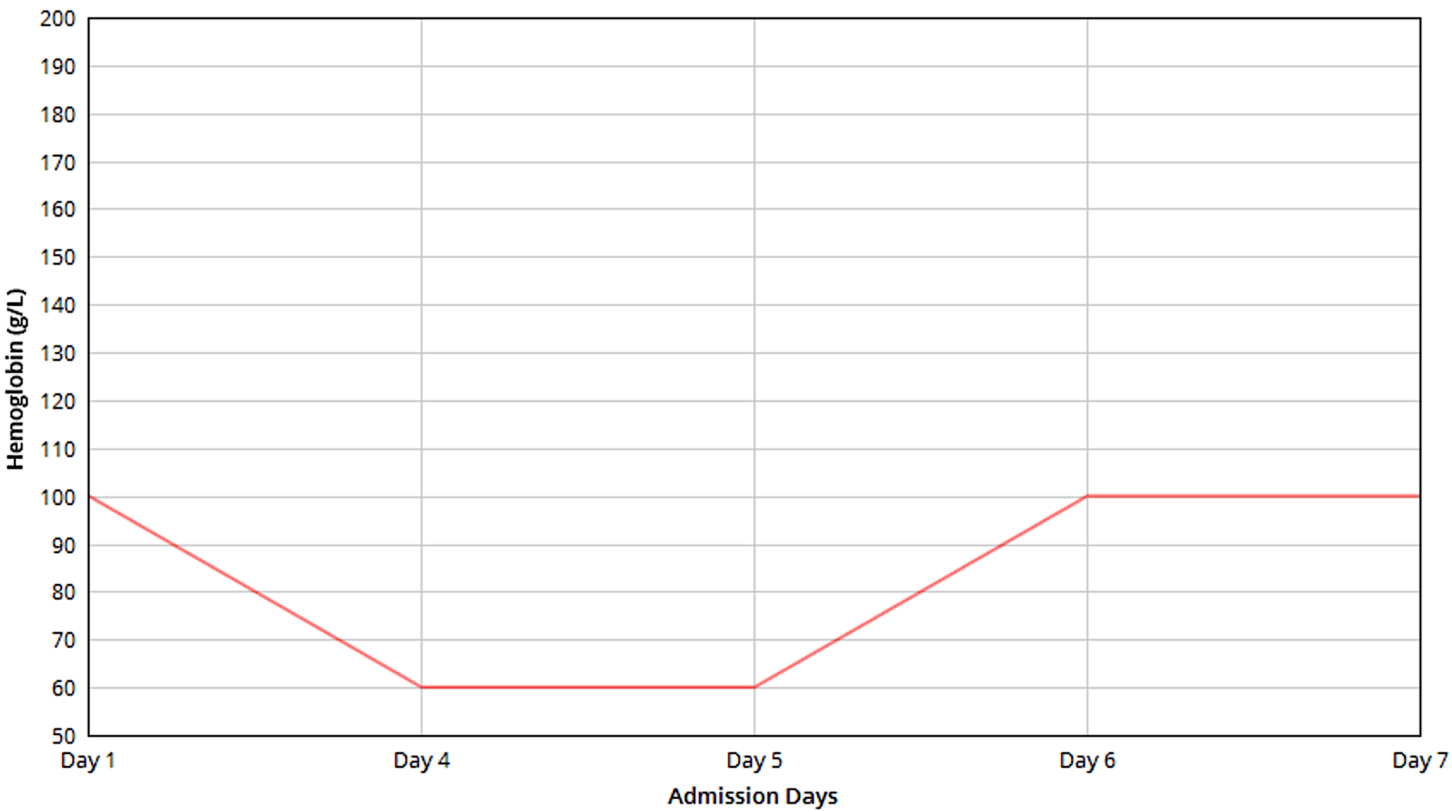
Hemoglobin Shows hemoglobin levels from Day 1, to Day 7, reflecting an initial decrease followed by a gradual improvement.

Hematocrit was 27%, and hemoglobin was 89 g/L, fluctuating throughout admission, as shown in Figure 2. 

**Figure 3:**
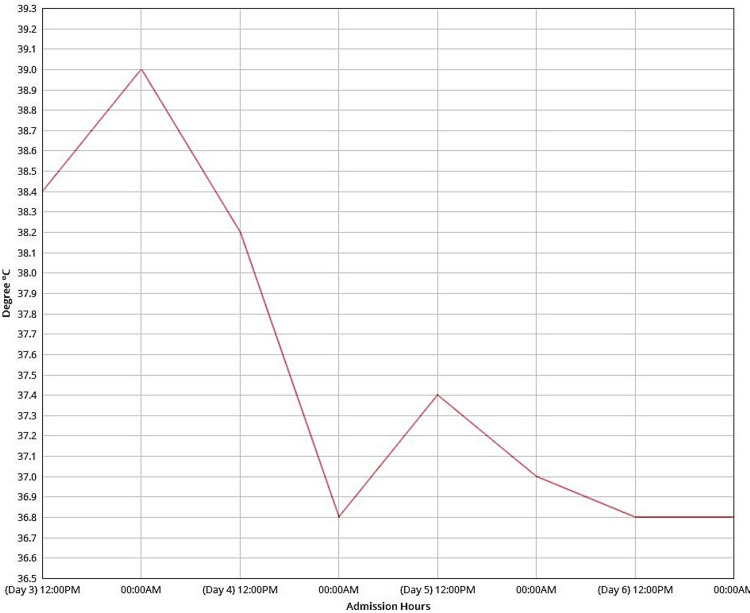
Tympanic temperature Temperature fluctuations over time, with an overall decline observed throughout the period.

Inflammatory markers showed a C-reactive protein (CRP) level of 12 mg/L and a procalcitonin level of 0.12 ng/mL. The coagulation profile indicated a prothrombin time (PT) of 12.2 seconds and an international normalized ratio (INR) of 1. Serum albumin was notably low at 28 g/L. Electrolytes, liver function tests, and renal function tests were within normal limits. Dengue PCR was positive, confirming a diagnosis of dengue fever. Further viral PCR tests for respiratory symptoms, including COVID-19, influenza, and respiratory syncytial virus (RSV), were negative. On Day 4, laboratory results indicated an increase in white cell count to 6.6×10⁹/L, a prothrombin time of 11.3 seconds, a partial thromboplastin time (PTT) of 34.3 seconds, and a CRP level of 69 mg/L. Tympanic temperature was measured throughout the admission period (Figure 3).

**Figure 4:**
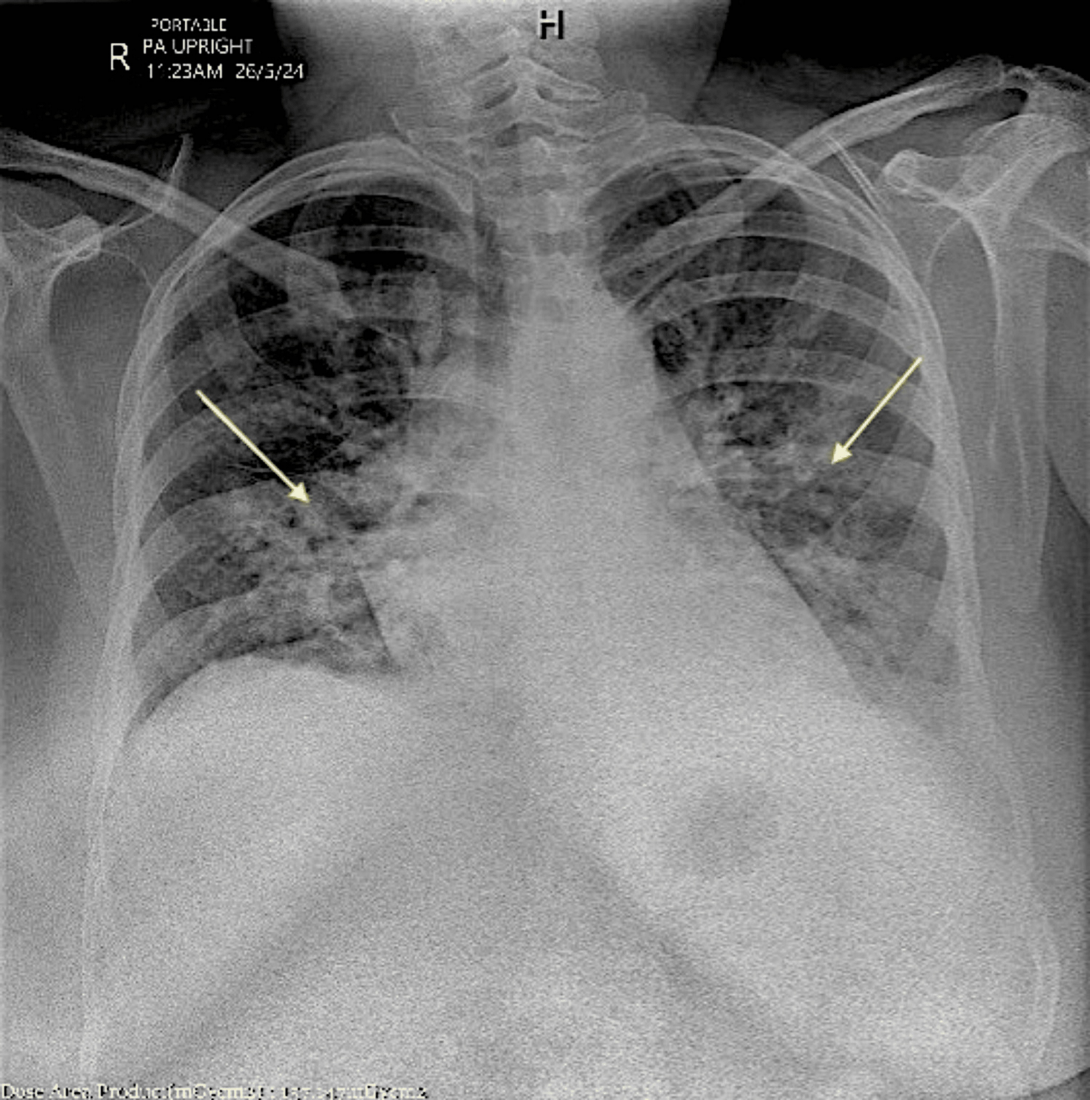
Chest X-ray Right-sided interstitial consolidation in the right lower lobe, with possible early interstitial consolidation at the base of the left lower lobe. Additionally, bilateral increased interstitial markings and perihilar bronchial wall thickening were observed, indicating infection; however, no pulmonary congestion was noted. The heart size and mediastinum were normal, and both hilar regions appeared unremarkable, showing no evidence of pneumothorax. (Yellow arrows are added to indicate the right-sided interstitial consolidation in the right lower lobe and likely early interstitial consolidation at the base of the left lower lobe, reflecting the presence of infection. The overall lung fields demonstrate bilateral increased interstitial markings, consistent with infection, while no pulmonary congestion or pneumothorax is observed.)

On Day 4, a chest X-ray was performed to evaluate the patient's persistent fever, hypoxia, and tachypnea (Figure 4). The Perc rule was applied to rule out pulmonary embolism for this patient, resulting in a score of three out of eight. The score, based on a heart rate over 100, unilateral leg swelling, and hormone use, prompted further evaluation for pulmonary embolism.

**Figure 5:**
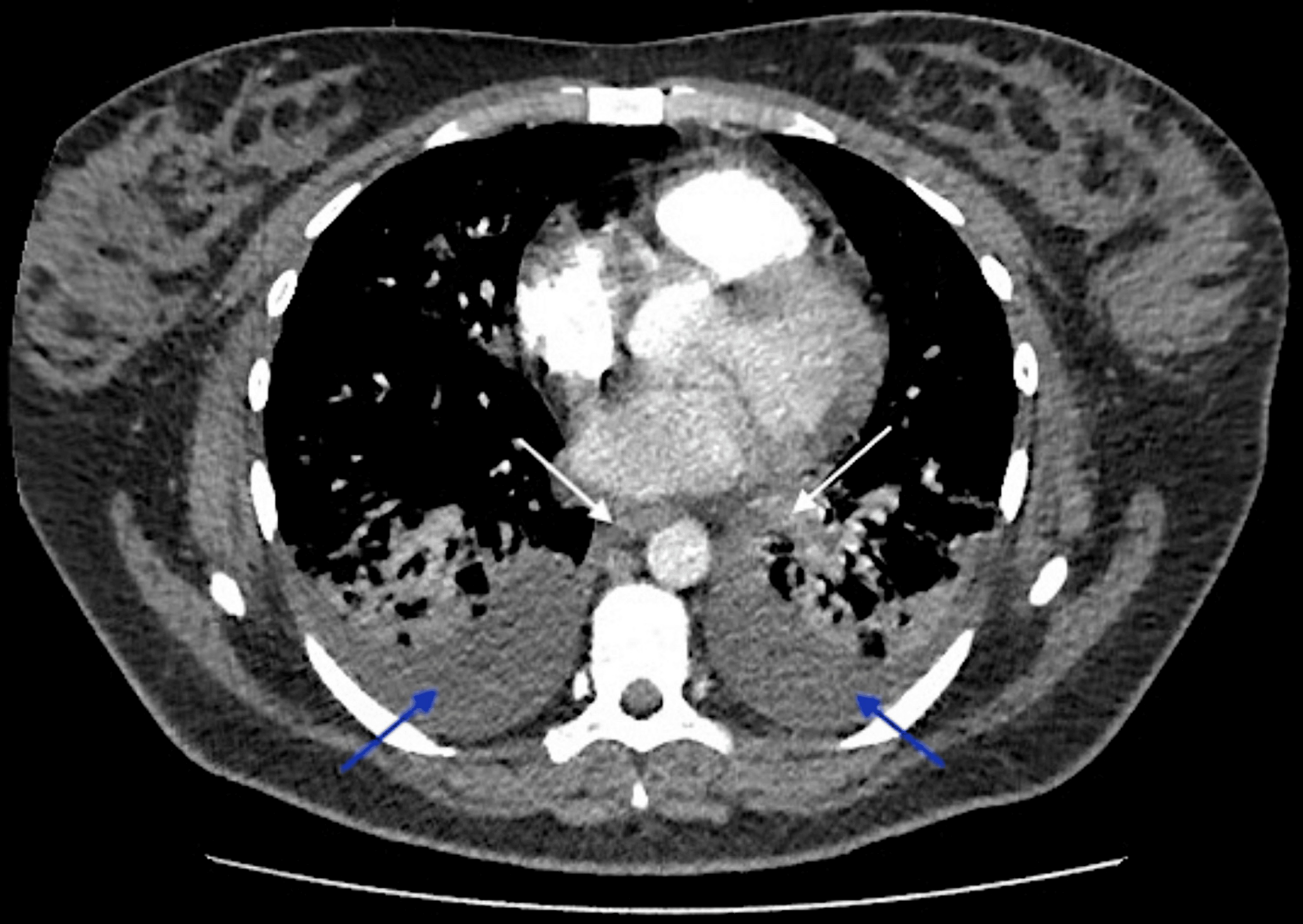
Chest CT scan Bilateral perihilar and centrilobular confluent consolidation along with small bilateral pleural effusions. Importantly, the pulmonary artery and its segmental branches were widely patent, showing no signs of pulmonary embolism or significant pericardial effusion. Additionally, there was no evidence of hilar or mediastinal lymphadenopathy, nor any notable bony abnormalities. These findings were consistent with bilateral bronchopneumonia and reactive effusion, effectively ruling out pulmonary embolism. (Arrows are added to highlight the bilateral perihilar consolidation located in the central part of both lungs near the hilum, annotated with white arrows. Additionally, the small bilateral pleural effusions are indicated at the lower posterior regions of both lungs, annotated with blue arrows.)

On the same day, a CT scan (Figure 5) was conducted to rule out pulmonary embolism. 

The patient was administered IV ceftriaxone and azithromycin, resulting in significant symptom improvement within 48 hours. Her dengue viral PCR test was positive, while the serology test was negative, and her inflammatory markers showed improvement. She was no longer hypoxic and ambulated without discomfort. The patient was scheduled for discharge on Day 6 with a prescription for oral antibiotics for outpatient completion, without deep vein thrombosis (DVT) prophylaxis, and continued aspirin as prescribed by her obstetrician. A follow-up appointment was arranged at the discharge clinic in one week.

## Discussion

Our report explored a rare complication of dengue fever in pregnant women. We present a case involving a 27-week pregnant patient who exhibited maternal pyrexia, tachycardia, vomiting, and abdominal pain, along with bilateral lower limb swelling. Laboratory findings revealed low hemoglobin and platelets, alongside a positive PCR for dengue, while tests for respiratory viruses (COVID-19, influenza, RSV) were negative. Chest X-ray indicated right-sided interstitial consolidation and a CT scan confirmed bilateral bronchopneumonia with reactive effusion, effectively ruling out pulmonary embolism.

A similar case involved a previously healthy young man diagnosed with dengue fever who was hospitalized due to pulmonary symptoms and later developed multiple lung cavitations. His treatment with ceftriaxone and oral cephalexin resulted in symptom improvement and reduced cavitation sizes [5]. This aligned with findings from a review that identified 26 case reports and 15 studies focused on bacteremia and bacterial coinfections in dengue, which noted that 0.18-7% of dengue cases presented with concurrent bacteremia, while bacterial coinfections are linked to 14.3-44.4% of dengue-related fatalities [6]. Another study assessing bacteremia in adults with prolonged dengue fever showed that 25% of patients had positive blood cultures, identifying bacteria such as *Staphylococcus aureus *and *coliforms*. Culture-positive patients displayed more severe symptoms, including intense body aches, higher fevers, and significant fluid accumulation. Notably, while white blood cell counts were ineffective in detecting bacteremia, low platelet counts and elevated C-reactive protein (CRP) levels were helpful indicators [7]. The identification of secondary infections is critical, and vigilance in recognizing and managing these infections is needed to improve patient outcomes in dengue cases.

A further notable example involves the rare concurrent infection of dengue fever with Legionella Pneumophila pneumonia, as reported in a 42-year-old man who developed severe respiratory distress, acute renal failure, and rhabdomyolysis. His condition worsened despite initial empiric treatment with ceftriaxone, but upon identification of Legionella through urinary antigen testing and treatment with azithromycin, the patient dramatically improved [8]. This case emphasized the complexity of managing bacterial superinfections alongside dengue, especially when patients present with rapidly progressing respiratory distress or septic shock.

The challenge of coinfections is exemplified by the concurrent presentation of dengue fever and COVID-19, which can complicate diagnosis due to overlapping clinical features. A case involving a 62-year-old man with fever, rash, and myalgia resulted in a dual diagnosis of COVID-19 pneumonia and dengue fever, confirmed by positive SARS-CoV-2 PCR and dengue serology. Despite underlying conditions such as diabetes, hypertension, and ischemic heart disease, he fully recovered with symptomatic treatment. This case underscored the critical importance of distinguishing between these infections, as misdiagnosis can delay treatment and elevate the risk of severe complications, including acute respiratory distress syndrome (ARDS) and hypovolemic shock [9]. It highlights the need for increased awareness of potential coinfections in patients with overlapping symptoms, reinforcing the significance of our study on the complexities of diagnosing dengue fever.

Dengue fever can lead to various clinical complications, including respiratory illness, as in our case. A recent case series from a center in Sri Lanka reported five patients with atypical dengue complications. Among them, a 53-year-old diabetic woman recovered from persistent shock, while a 34-year-old woman was treated for sinus bradycardia. A 23-year-old woman with severe plasma leakage received intravenous albumin, and a 34-year-old pregnant woman required an emergency cesarean section. Tragically, a 27-year-old woman with hemophagocytic lymphohistiocytosis succumbed to multiorgan failure [10]. These cases highlight the significant risk of complications associated with dengue fever, particularly for vulnerable populations such as pregnant women and those with underlying health conditions.

Pregnancy could be considered a risk factor for complicated dengue cases. A study reviewing ICU patients with severe dengue and concurrent bacteremia identified that 15.5% had bacteremia, primarily due to *Streptococcus*, *E. coli*, and *Staphylococcus*. Patients with bacteremia had higher mortality rates (40.9% vs. 18.3%), elevated CRP levels, and longer activated partial thromboplastin times, highlighting these as significant risk factors for bacteremia in severe dengue patients [11]. Additionally, diabetes should be considered when prioritizing and closely monitoring patients during dengue outbreaks, as there is a significant association necessitating early dengue confirmation in diabetic patients in endemic regions. Timely intervention is crucial to prevent serious complications and death in those with acute dengue [12]. Other factors include age (young children and elderly), female sex, genetic predisposition, immune status, viral strain virulence, malnutrition, and co-morbidities(e.g., diabetes, hypertension, and chronic kidney disease). Additionally, environmental factors play a significant role in shaping the spread and severity of dengue [13]. Early detection and prompt management were crucial in reducing morbidity and mortality in these high-risk groups.

The case also parallels another report of a two-and-a-half-year-old girl with dengue fever presented with fever, cough, and respiratory distress, leading to the diagnosis of necrotizing pneumonia. Despite initial treatment, her condition deteriorated, requiring intensive care and broad-spectrum antibiotics [14]. This case highlights the complexities of managing pneumonia in dengue patients, emphasizing the need for careful monitoring and timely intervention to address potential bacterial superinfections and the challenges posed by overlapping clinical symptoms. Further research is warranted to improve understanding and treatment strategies for these concurrent infections. 

In considering the pneumonia's etiology, it is likely related to dengue fever rather than being nosocomial, as the onset of pneumonia coincided with dengue symptoms. Furthermore, it is believed that DENV may induce immunosuppression, potentially resulting in concurrent bacterial infections. The primary risk factor for *Staphylococcus aureus-*related healthcare-associated pneumonia is endotracheal tube ventilation, which was not utilized in this case. Given the low incidence of this pneumonia type, dengue infection is regarded as the primary risk factor, underscoring the need for further research in this area. This study has limitations, including a small sample size and potential for selection bias, which may impact the generalizability of the findings.

## Conclusions

In conclusion, this case report discussed a rare presentation of dengue fever complicated by bacterial pneumonia in a pregnant patient, emphasizing the importance of considering dengue in patients with fever, respiratory symptoms, and hematologic abnormalities, especially in endemic regions. Early recognition and prompt management are crucial to improving outcomes. Collaboration among healthcare professionals is essential for accurate diagnosis and treatment, particularly with complications like pneumonia. Future research is needed to address the remaining uncertainties regarding dengue fever's complications and to further clarify the relationship between dengue and secondary infections.
